# Proteomic Markers for Mechanobiological Properties of Metastatic Cancer Cells

**DOI:** 10.3390/ijms24054773

**Published:** 2023-03-01

**Authors:** Sergey Leonov, Olumide Inyang, Konstantin Achkasov, Elizaveta Bogdan, Elizaveta Kontareva, Yongheng Chen, Ying Fu, Andreyan N. Osipov, Margarita Pustovalova, Yulia Merkher

**Affiliations:** 1 School of Biological and Medical Physics, Moscow Institute of Physics and Technology, 141700 Dolgoprudny, Moscow Region, Russia; 2 Institute of Cell Biophysics, Russian Academy of Sciences, 142290 Pushchino, Moscow Region, Russia; 3 Department of Oncology, NHC Key Laboratory of Cancer Proteomics & State Local Joint Engineering Laboratory for Anticancer Drugs, National Clinical Research Center for Geriatric Disorders, Xiangya Hospital, Central South University, Changsha 410008, China; 4 State Research Center—Burnasyan Federal Medical Biophysical Center of Federal Medical-Biological Agency, 123098 Moscow, Russia; 5 N.N. Semenov Federal Research Center for Chemical Physics, Russian Academy of Sciences, 119991 Moscow, Russia

**Keywords:** cancer, metastasis, invasion, endocytosis, mechanobiology, mechanotypes, bio-markers

## Abstract

The major cause (more than 90%) of all cancer-related deaths is metastasis, thus its prediction can critically affect the survival rate. Metastases are currently predicted by lymph-node status, tumor size, histopathology and genetic testing; however, all these are not infallible, and obtaining results may require weeks. The identification of new potential prognostic factors will be an important source of risk information for the practicing oncologist, potentially leading to enhanced patient care through the proactive optimization of treatment strategies. Recently, the new mechanobiology-related techniques, independent of genetics, based on the mechanical invasiveness of cancer cells (microfluidic, gel indentation assays, migration assays etc.), demonstrated a high success rate for the detection of tumor cell metastasis propensity. However, they are still far away from clinical implementation due to complexity. Hence, the exploration of novel markers related to the mechanobiological properties of tumor cells may have a direct impact on the prognosis of metastasis. Our concise review deepens our knowledge of the factors that regulate cancer cell mechanotype and invasion, and incites further studies to develop therapeutics that target multiple mechanisms of invasion for improved clinical benefit. It may open a new clinical dimension that will improve cancer prognosis and increase the effectiveness of tumor therapies.

## 1. A Need for Identification of New Potential Prognostic Markers

Today, cancer is the second cause of death worldwide [1]. There are different methods to detect cancer, such as blood tests with cancer-specific markers, imaging (includes MRI, CT, X-ray, ultrasound techniques) and endoscopy. However, the major cause (more than 90%) of all cancer related deaths is metastasis [2,3], thus its prediction and efficient cure can critically affect the survival rate. Currently, the choice of treatment tactics for non-small cell lung cancer (NSCLC) and triple negative breast cancer (TNBC) depends on the stage of the disease and the general condition of the patients. Although lymph-node status, tumor size, histopathology and genetic testing currently predict metastases, not all of these are infallible, and obtaining results may require weeks. For example, in breast cancer, lymph nodes that have reached metastases are commonly present, yet about 30% of patients with negative lymph-node status also develop metastases [4]. The TNM classification, considering the size of the primary tumor and metastasis to lymph nodes or distant sites, provides some prognostic prediction of the outcome even though the classification does not consider the organs of metastatic growth [5]. An additional technique for metastatic cancer prognosis is genetic testing. It allows for the identification of specific subtypes within an overall disease category based on differences in gene expression [6]. Unfortunately this technique has a limitation in that the testing can only provide information on specifically identified genes and in specific cancers (for example, pancreatic cancer [5]) and cancer mutation prognostic markers are still undetermined. In practice, the sensitivity and specificity of individual markers may vary widely and there are a number of physiological and pathological factors that can affect the results [7]. The clinical gold-standard histopathology examination is qualitative and based on cancer-type statistics, wherein the regular (i.e., non-urgent) histological grading of tumor samples entails tissue fixation and usually takes several days to weeks. The automated analysis of histopathology images of fixed tissue can accelerate results [8], yet accuracy is challenging and even novel deep-learning approaches have achieved at most 67% agreement with manual histopathology [9]. Thus, pathological grading has been constrained by longer timescales and uncertainties in the prognosis of metastatic likelihood, and this can cause substantial distress in patients and can degrade their immune defense and healing [10]. 

**Figure 1:**
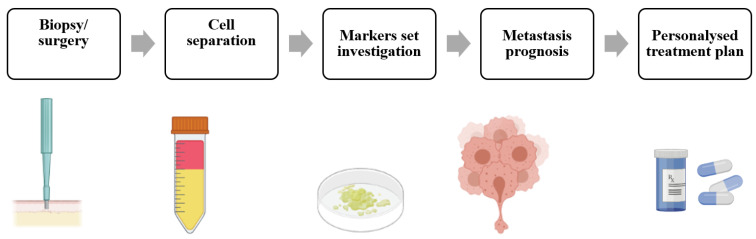
Potential approach for novel theragnostic markers.

The identification of new potential prognostic factors will be an important source of risk information for the practicing oncologist, potentially leading to enhanced patient care through the proactive optimization of treatment strategies (Figure 1). Recently, the new techniques, independent of genetics, based on the mechanical invasiveness of cancer cells (microfluidic, gel indentation assays, migration assays, etc.), demonstrated a high success rate for metastasis detection in research labs [11]; however, due to complexity, they are still far away from clinical implementation. Summarizing all the limitations listed above, new approaches are highly required for the accurate estimation of the risk for metastasis, preferably with results provided rapidly, in quantitative measures and independent of user bias.

## 2. Tumor Biomarkers 

Cancer cells display a wide range of genetic alterations that include point mutations, gene amplification, and gene rearrangements, which disrupt molecular pathways that control growth, survival, and metastasis [12]. However, changes in the molecular signatures may reflect hyperproliferation, genotoxicity, altered gene expression patterns, hyperplasia, and enzymatic changes that respond to inherited and environmental causes of cancer. Therefore, biomarkers for cancer detection should be carefully examined. Biomarkers are valuable indicators in cancer detection because they possess a unique molecular signature secreted by a cell becoming neoplastic or a specific body response to cancer, measurable in cells, tissues or fluid [13]. They are cellular, biochemical, and molecular (proteome, genetic, and epigenetic) alterations that can be used to identify or monitor a normal, abnormal, or biological process. They may evaluate normal biological processes, pathogenic processes, and pharmacologic reactions to treatment intervention, and they are subject to change during pathological conditions. Hence, they may indicate the physiological state of a cell. Cancer is detected using different biomarkers such as genes and epigenetic markers, metabolic, cancer proteomics, etc. [14]. Cancer gene mutation testing is used to determine the absence or presence of specific inherited mutations in genes known to play significant roles in cancer development. For example, blood tests to identify BRCA1 and BRCA2 gene mutations, which indicate the development of cancer in the breast [15]. 

Cytogenetic and cytokinesis markers are classical cancer markers used to identify structural and numerical aberrations in the chromosomes because of the association between chromosomal aberrations and neoplastic transformation. Structural aberrations in malignant tumors, which are easily scored using various banding techniques, arise from hyper- and hypo-diploid, aneuploidy, sister chromatid exchanges, and translocation caused by deviation in the diploid number of chromosomes [16]. An example of a cytogenetic marker is somatic mutations in reporter genes, oncogenes, and tumor suppressor genes, which are promising biomarkers for cancer risk because of their ability to capture genetic events associated with malignant transformation [14]. Enhanced glucose utilization is a fundamental change in many tumor cells regardless of their histological origin and nature of mutations [17]. This is because the rapidly dividing cancer cells have high demands for energy and nutrients for their metabolic process. As a result, changes in the tumor environment, the inactivation of tumor suppressor genes and the activation of oncogenes cause aberrant glucose metabolism [18]. Hence, glucose utilization is a useful metabolic marker to diagnose cancer [19].

Many proteins and other macromolecules secreted into the extracellular milieu by cancer cells serve as biomarkers. Some of these secreted proteins enter the bloodstream and serve as antigen-based biomarkers in the serum. Some important cancer antigens used for diagnostic and prognostic biomarkers are prostate-specific antigens (PSA) in prostate cancer, alpha-fetoprotein in hepatocellular carcinomas, cancer antigen 15-3 in breast cancer, etc. [14]. Tumor cells circulating in biological fluids are also used as diagnostic biomarkers as they help to capture, identify and count tumor cells inside the human body. This is possible because these cells detach from solid tumors and enter the bloodstream [14].

Much attention has been paid recently to the role of proteomics markers in theragnosis. Several identified protein biomarkers are in use clinically to monitor both disease progression and therapeutic efficacy [20]. For example, PSA is a well-known biomarker for prostate cancer, and its diagnostic usefulness is well-established [21]. Another example is the HER2 marker for breast cancer, when HER2-positive cancers tend to grow and spread faster than HER2-negative cancers [22,23]. Specific molecular and genetic markers have been established previously [24]. Proteomics has emerged as a promising field in the post-genomic era. Proteomics can provide much more information at the cellular function level than genomics and transcriptomics, and there is a poor correlation between protein expression and the copy number of genes in cancer cells [25]. Most recently, mass spectrometry-based proteomics techniques have been at the forefront of cancer research and biomarker discovery studies. The analytical capabilities of many MS-based proteomics strategies have increased in terms of sensitivity, specificity and accuracy, facilitating the analysis of several thousand proteins rapidly and accurately in a single study. In the previous study, Tenascin-C was identified [26] as a potential stromal biomarker for colorectal cancer metastasis by the combination of laser capture microdissection (LCM), iTRAQ labeling and two-dimensional liquid chromatography-tandem mass spectrometry (2D LC-MS/MS) methodologies. 

## 3. Mechanobiology of Metastasis

Altered mechanotype is an emerging hallmark of cancer cells that is linked to invasive phenotype and treatment resistance. Mechanotype also influences crosstalk between tumor cells and their environment and may thus have a critical role in cancer progression. Tumor cell mechanotype appears to relate to invasive status [27,28,29]. Demonstration that the invasive potential of cells correlates with their deformability, where softer or more deformable cells are more invasive, sounds plausible, since more deformable cells can move more easily through tight gaps, which could assist their escape from a primary tumor and invasion into surrounding ECM [27,29]. In vitro experiments have shown that malignant cancer cells are softer than benign cells. This can be shown through experiments on human cell lines derived from various tissues using different mechanotyping methods. Atomic force microscopy [30,31], deformability cytometry [32] and parallel microfiltration [28] are among the mostly widely used. Many of these methods measure the displacement or change in shape of a cell or protein network. Atomic force microscopy is used to measure the viscoelastic properties of a single cell or protein network with displacements down to 1 nm. Other methods can probe mechanical properties over length scales of 1–10 um, such as magnetic twisting cytometry and micropipette aspiration. The deformability of whole cells can also be measured by forcing cells to pass through smaller pores than cell size. As such, the parallel microfiltration method, where air pressure forces a cell suspension to pass through a porous membrane, can measure the relative deformability of different cell samples [28]. Microfluidic deformability cytometry measures whole-cell deformability by applying stretching extensional flow to single cells [32]. Active forces generated by the cell can be probed by traction force microscopy, where displacements of the substrate that result from contractile forces of the cell are measured. 

Cell deformability is associated with the aggressiveness of tumor cells. Indeed, overexpression of key epithelial-to-mesenchymal transition transcription factors (Snail, Slug and Zeb1) makes ovarian cancer cells softer [28]. Human MCF-7 cells are more deformable than their non-malignant mammary epithelial counterparts, MCF-10 cells. Metastatic MCF-7 (modMCF-7) cells are even more deformable than the less-invasive MCF-7 cells [27]. Human lung adenocarcinoma cells with greater metastatic potential are also more deformable than their less-metastatic counterparts [29]. Human bladder epithelial cancer cells are more deformable than normal cells [33]. Similarly, transformed fibroblasts are significantly more deformable than normal untransformed fibroblasts [27,34]. Taken together, malignant cells across various types of cancers are more deformable than normal cells. Moreover, more-invasive tumor cells are softer or more tissue-compliant than less-invasive ones.

It is a complex and challenging task to understand the molecular roots of malignant cells’ altered mechanotypes. Mechanotypes can change through multiple factors including proteins, signaling pathways and other factors. Changes to cytoskeleton network structure and organization can alter cell differentiation or deformability. Such structural changes are also linked to malignant phenotypes. Higher grade colon and ovarian cancer cells have more actin and microtubule content than lower grade cancer cells [35,36]. Differences in the cytoskeletal architecture are also involved in variations in the deformability of melanoma cells; these structural alterations are associated with in vivo metastatic potential in mouse models [37]. However, the cytoskeleton changes in a cancer cell do not always correlate with the softer mechanotype. Even though softer mesenchymal-type ovarian cancer cells are often found less readily than their epithelial-type counterparts, there is no uniform pattern of actin distribution or microtubule organization that can explain the softer mechanotype [28]. Thus, the cell mechanotype provides unique information about the malignant status of a cell, being a good candidate for a physical biomarker of malignancy.

Metastatic invasion through tissue is a critical step in metastases formation. The most widely used measure of cancer aggressiveness is cell invasiveness, or the ability of a cell to invade its surroundings. Migration is often used to describe any directed cell movement within the body, while *invasion* is defined as the penetration of tissue barriers [38]. In vitro methods enable the study of confined cell migration in environments of known physical and chemical composition [39]. A simple method for the migration measurement of single cells is colloidal gold particle-coated surfaces, with areas of clearing in the gold-colloid corresponding to phagokinetic cell tracks [40,41]. Microfluidic and nanofluidic assays, with relatively rigid or compliant channels fabricated from silicon or poly(dimethyl siloxane) (PDMS), were also employed to simulate the flow of single cells through blood or lymph vessels [42,43] or transition effects across mechanical barriers [44]. The enhanced pliability of the metastatic cancer cells facilitates their migration through small pores, as was evaluated by in vitro Boyden chamber migration/invasion experiments [45,46]. Boyden chambers were previously evaluated as a commonly employed migration assay that measures the capacity of cell motility and invasiveness toward a chemo-attractant gradient through a porous matrix [46,47]. However, the experiments last at least 24h and there is dependence on Matrigel batches: for example, it was found that in the same experiment MDA-MB-231 cells seeded on two different batches of Matrigel resulted in, respectively, 23 ± 2% and 15 ± 2% invasion [48]. It was previously shown by example of Boyden chambers (matrix membranes with different mesh sizes), that the structure and mechanics of cancer cells are linked directly to their metastatic potential [45,46]. However, the results are affected by the thickness and composition of membranes, and the invasion capacity of cells with different phenotypes cannot be universally correlated to metastatic potential [48]. Therefore, before each experiment, the chosen standard should be checked. There are some complex 3D invasion gel assays for in vitro invasion; they usually utilize a degradable collagen [49] or gelatin [50,51] matrix and most faithfully mimic the situation in vivo, however, they require complex equipment such as a confocal microscope or fixation and sectioning [38]. The novel, mechanobiology-based, simple 2D gel-invasion assay provides relatively fast (one day), quantitative results, with very high accuracy [52,53,54]. Recently, we have shown the direct connection of the ability of metastatic cells to invade with their propensity to endocytosis, and have linked the efficiency of short-time (1 h) encapsulation of nanoparticles to the metastatic potential [55,56]. However, those approaches are still far away from clinical implementation, due to the required manipulations for cancer cell extraction from tumor samples. The definition of specific protein markers, reflecting the direct mechanobiological properties of cancer cells (i.e., invasive and migration properties), will allow the detection of metastatic potential with no need for intact cell extraction from the tumor sample, and therefore will provide an important input in the prognosis of metastasis [57].

## 4. Invasion and Endocytosis

Invasion is a key event towards the acquisition of the metastatic phenotype by tumor cells and an attractive target for anticancer therapy aimed at the prevention of metastasis. During the invasion process, cancer cells change their shape and apply forces to their surroundings. The internal environment of highly metastatic cells is more dynamic, and their cytoskeleton is sparser than both low metastatic potential (MP) and benign cells, and the high MP cells are also more receptive to internalizing materials from their surroundings. It was previously shown that the ability to migrate, structure and mechanics of cancer cells are factors directly linked to their metastatic potential [46,53].

**Figure 2:**
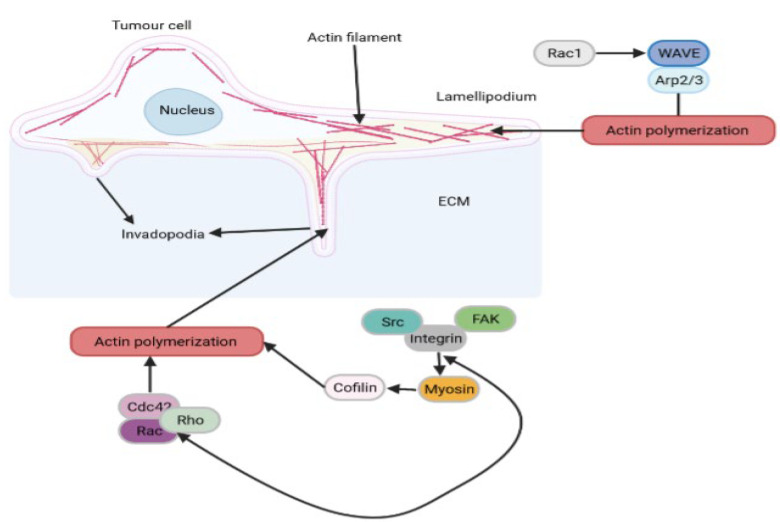
Intracellular mechanisms of invading cancer cell.

Cell migration begins with the expansion of cell membrane protrusions, which are powered by an uninterrupted cycle of actin polymerization and depolymerization (Figure 2). Upon adhesion to the ECM via integrin- and FAK-containing complexes and actin–myosin II-mediated cell contraction, adhesion release at the trailing edge results in cell motility. During this process, the cofilin pathway operates as the “steering wheel of the cell” by coordinating membrane protrusion [58]. The protrusion of lamellipodium is supported by the continuous growth of actin filaments toward its leading edge [59]. Actin-related protein 2/3 (ARP2/3) complex, which is the major actin nucleator in lamellipodia, generates a new actin filament that branches off the side of a pre-existing filament [60]. The ARP2/3 complex upon activation by small GTPase Rac and WASP-family verprolin homologous NPF proteins such as WAVE1, WAVE2 and WAVE3 mediates actin polymerization within lamellipodia and ruffles, and in turn promotes cell migration. During actin polymerization, the actin network and ARP2/3 complexes form branched junctions and experience a retrograde flow with respect to the plasma membrane, while the elongators remain dynamically associated with the plasma membrane during their entire active phase. The retrograde flow is as a result of the combined action of actin polymerization at the cell leading edge, which pushes the lamellipodial actin network backwards, and the myosin contraction at the back of the lamellipodia that pulls the lamellipodial actin network backwards [61]. The persistence of the lamellipodial actin network thus emerges as a critical factor in steering cell migration. Podosomes and invadopodia (collectively known as invadosomes) are areas of increased actin polymerization [62], and they facilitate invasion. The structure of the invadosome is made up of two repeating polymerizing actin arrangements characterized by lengthy pillars of densely coiled F-actin filaments positioned perpendicular to the substrate. This structure is known as the nucleus of the invadosome structure because it forms an actin cluster composed of radial F-actin filaments parallel to the substrate [63]. As a result, a dense F-actin core surrounded by a closed ring of adhesion molecules that colocalize on the actin cluster represents a single invadosome. Extracellular matrix receptors, including CD44, β1, β3, and β5 integrins, are associated with invadosomes [64,65]. The associated receptors within invadosomes sustain the localization of many adaptor proteins, including those found in focal adhesions comprising tyrosine kinases such as FAK, Pyk2, Src, and small GTPases. Among these small GTPases are Cdc42, Rac and Rho, and adaptor molecules, such as p130Cas, paxillin, and vinculin. Finally, invadosomes essentially perform two major functions: exerting actin-reach and adhesive cellular protrusions or components and governing polarized secretory signaling pathways that maintain the precisely controlled supply of metalloproteases required for extracellular matrix degradation [66]. 

The regulated assembly of actin filament networks plays an essential role in endocytosis. It also accounts for many of the mechanical properties of the cytoplasm. Nucleation, an initial step of actin filament formation, is a process that involves the combination of actin monomers and is an essential stage of actin regulation. Actin filament-nucleating proteins such as Arp2/3 seed a few actin filaments near the endocytic pit and promote actin nucleation by binding with these mother filaments in the presence of nucleation promoting factors (NPFs) [67,68]. The Arp2/3 complex, which is naturally inactive, is activated by activator proteins or NPFs, such as the Wiskott–Aldrich syndrome protein and cortactin, among others [69]. About 200 activated Arp2/3 complexes are said to assemble at sites of clathrin-mediated endocytosis in human cells, supporting robust internationalization. These complexes assemble actin filaments at the sites of endocytosis. Actin self-organizes into a radical branched array with growing ends oriented toward the base of the pit. The long actin filaments bend between the attachment sites and the pit’s base. The elastic energy stored in these bent filaments contributes to endocytic internalization [67]. This force generated by actin assembly is adequate to deform cell membranes and move particles within dense cytoplasm. The arrangement of these filaments around the endocytic sites determines the exact mechanism for the role of actin in endocytosis [70]. First, endocytic proteins with clathrin as the first protein to emerge are recruited to endocytic sites, and later on, proteins that regulate the assembly of actin appear. Clathrin is the protein to be recruited, after which proteins of the endocytic machinery, as well as the regulators of actin assembly, are recruited. WASp-interacting protein (WIP/Vrp1), and Myo5, a type-I myosin, are recruited. Next, the inward movement of the endocytic patch takes place, which first involves actin polymerization marked by the appearance of the Arp2/3 complex, actin, most other actin-binding proteins [71,72] and the BAR-domain amphiphysin proteins such as Rvs161 and Rvs167, and then involves the initiation of inward movement [73]. Actin nucleation then takes place on the endocytic vesicle membrane during invagination as well as when the vesicle moves away from the plasma membrane. After the completion of this short movement into the cytoplasm, all the endocytic proteins leave the vesicle. Membrane fission allows the endocytic vessel/actin patch to move about the cytoplasm. These actin patches make longer-range movements in and about the cytoplasm [74]. The signaling receptor tyrosine kinase (RTKs) causes receptors to be recruited to internalization-devoid structures such as coated pits, where they are internalized through a process that majorly depends on Rab5. Clathrin-dependent and -independent routes mediate receptor internalization. Internalized receptors are then transported to early endosomes (EEs). These early endosomes, which consist of small vesicles and tubules, are fused with endocytic vessels, facilitating the dissociation of many ligands from their receptors. The newly freed ligands then pile up in the EE lumen where they are transported to the late endosomes and finally to the lysosomes where they are degraded. Through the subsequent action of various endosomal sorting complexes required for transport (ESCRT), the receptors are internalized into multivesicular bodies and finally transported to lysosomes for degradation. ESCRTs are therefore agents of cargo selection and vesicle formation. Receptors could be recycled back to the plasma membrane instead of undergoing degradation. This is possible by either returning from EEs or passing through a population of pericentriolar organelles known as recycling endosomes [75,76,77]. 

**Figure 3:**
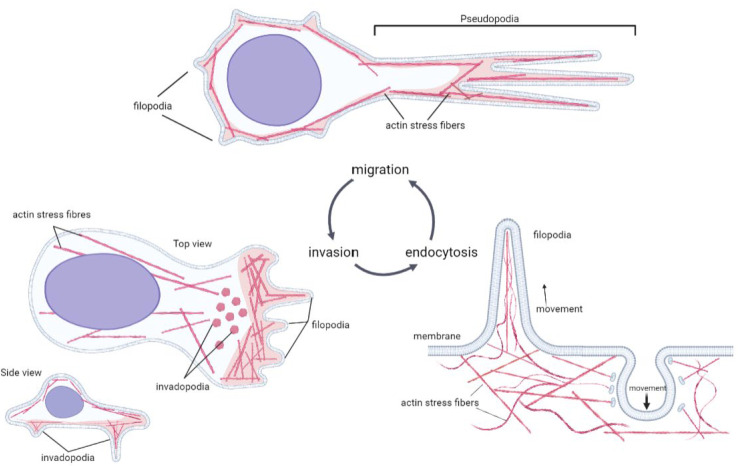
Actin cytoskeleton remodeling in invasion, migration and endocytosis—schematic diagram.

The cytoskeleton machinery mechanisms (i.e., actin network), utilized by metastatic cells for the invasion process, have been widely studied and found to be similar to the involvement of the actin cytoskeleton in regulation endocytosis pathways [78] (Figure 3). The great recovery ability and plasticity of highly invasive metastatic cells are related to the actomyosin contractile apparatus and to actin remodeling [79]. Actin cytoskeleton remodeling is a crucial mechanism for cell invasion during metastasis that is mediated by the Rho GTPases. Actin also plays an obligatory role in endocytosis—actin was proposed to participate at multiple stages of endocytosis, including membrane invagination, scission and propulsion of the endocytic vesicle [70]. Actively remodeling actin network cells are able to successfully facilitate endocytosis [80]. Therefore, there is a direct connection between the ability of cancer cells to invade and endocytosis propensity. Cell mechanobiology approaches for cancer diagnosis and prognosis, with the main focus on understanding metastasis formation and progression [53] and developing patient-specific platforms to predict the likelihood of metastasis formation [52], have been previously developed. The approaches allowed for determining the critical role of actin in the invasion and migration of metastatic cells [81]. We have previously demonstrated that highly metastatic cells are able to encapsulate, during a short period of time (1h), significantly more nanoparticles than cells with low metastatic potential [55,56]. The ability to define proteotype-based markers connecting invasiveness, endocytosis propensity and thus metastatic potential is highly important for metastasis prediction and treatment. The features of cancer cells endocytosis are regulated by the same protein and genetic mechanisms, which are involved in cancer development. The overall similarities of intracellular processes between invasion and endocytosis are summarized in Table 1. The sufficient amount of such similarities leads us to the hypothesis that there is not only the likeness in cellular mechanics between endocytosis and invasion, but also shared markers for both processes.

## 5. Proteins Involved in Actin Cytoskeleton Remodeling

Proteins involved in actin cytoskeleton remodeling play an important role in the mechanisms of tumor cell migration and invasion [105]. The WAVE2 protein is involved in actin filament reorganization and lamellipodia formation and was shown to colocalize with Arp2 at the invasive front of breast cancer [105,106]. Cortactin regulates cortical actin cytoskeleton dynamics by stabilizing F-actin networks and promoting actin polymerization via activating the Arp2/3 complex [107,108], which is actively employed by the mechanism for cortactin-mediated endocytosis [109]. The polymerization of phosphorylated cortactin and actin at aggressive pseudopodia increases the invasiveness of human breast cancer cells and subsequently induces matrix degradation and aggressive behavior [110]. From the other side, cortactin and dynamin-2 coordination and dynamic interaction may provide a mechanical force responsible for the actin assembly-driven movement of endocytic vesicles to the deep cytosol, eventually leading to vesicle detachment from a protrusive membrane [111]. Dynamin-2 is a widely expressed large GTPase identified for its pivotal role in endocytosis and intracellular membrane trafficking and cytoskeleton regulation. DNM2-dependent processes in cancer cells have been described, explaining its impact on cancer pathology. DNM2 dysfunction can promote cell migration, invasion, and metastasis [112]. Markers for Arp2/3 complexes are the major actin nucleators and can provide diagnostic opportunities in cancer [24]. Arp2/3 deregulation promotes cancer progression [113] and is important in endocytosis [78]. The ARP2/3 complex, upon activation by small GTPase Rac and WASP-family verprolin homologous NPF proteins, mediates actin polymerization within lamellipodia, invadopodia and ruffles, and in turn promotes cell migration and invasion [60,114]. In combination with this, Arp2/3 is essential and conserved in clathrin-mediated endocytosis, and allows endocytic vesicle invagination, maturation, and ultimately scission through a stepwise process of pulling, sculpting, and pushing [67]. The MENA protein regulates actin polymerization and cell migration. An elevated level of the MENA in isoform, which is involved in the formation of invadopodia due to the phosphorylation of cortactin and activation of the N-WASP/Arp2/3 complex, is found in invasive cells of human tumors and animal tumor models and is associated with a high risk of metastasis [115]. Additional common markers for invasiveness are: FRA1—overexpressed in breast and lung cancers and is associated with biological functions such as tumor proliferation, differentiation, invasion and apoptosis [116] (the marker was studied and characterized in our laboratory); ZEB1—present in both lung and breast cancers and is a master regulator of the EMT program and also associated with tumor invasiveness and metastasis [117]; DNA-PK, CD44, CD166—shown by us and others to be active contributors for metastasis. Vimentin was shown as a promoter of directed cell migration by coordinating the dynamics of actin filaments and microtubules [118]. Caveolin-1 (CAV-1), an integral membrane protein, is highly expressed in triple-negative BC cells and has been reported to promote proliferation [119]. The interaction between Cav-1 and Rho-GTPase promotes metastasis by elevating the expression of α5-integrin and enhancing the activation of Src, Ras and Erk [120]. Caveolin-1 regulates the formation of caveolae, an invaginated membrane structure in the membrane endocytic system with different transport and sorting functions, and therefore is responsible for caveolin-mediated endocytosis [121]. Palladin is another actin-associated protein, and its overexpression correlates with the invasive motility in human breast cancer cells [122]. Palladin serves as a cytoskeleton scaffolding molecule by interacting with various actin-binding proteins essential for cytoskeleton organization via podosome activation [123]. Swiprosin-1 regulates lamellipodial membrane dynamics as an actin-binding and bundling protein, directing cell protrusion and enhanced migration via the activation of the Rho family of proteins, including Rac1, Cdc42 and RhoA. On the other hand, Swiprosin-1 was identified as a cargo-specific adaptor for bridging the clathrin- and dynamin-independent endocytic machinery to Rab21-bound integrins, and it couples Rab21 endosomes and their motility in cells to the actin cytoskeleton [124]. The signalling pathways relevant to cell invasion and migration and their potential clinical biomarkers are summarized in Table 2.

Recently, a list of 76 potential candidates (out of 1245 mentioned in the literature) for biomarkers of cancer invasiveness was established at the crossroads between the literature data and experimental and clinical data [20]. Among this list, we decided to choose 10 potential candidates that are directly related to actin cytoskeleton remodeling and have not been widely studied (Table 3). Annexin A6 and A2 are presented in different types of cancer. They are secreted via the exosomal pathway and have a proven role in cell migration by the formation of reversible, membrane-cytoskeleton complexes through interactions with actin and α-actinin. Extracellular matrix protein 1 (ECM1) is significantly elevated in a number of epithelial tumors and invasive breast cancer, giving rise to metastases. Transgelin is a protein that affects the dynamics of the actin cytoskeleton through the stabilization of actin filaments. Transgelin positivity has been associated with more aggressive tumors. Profilin-1 (PROF1) is an actin-monomer binding protein. It regulates actin dynamics and cell motility and plays an important role in the migration of cancer cells. It was associated with aggressive clinic-pathological characteristics and a poor prognosis. Myosin 9 is a cytokine, involved in cytoskeleton reorganization. It plays an important role in the formation of cellular pseudopodia and is closely related to the progression and poor prognosis of the majority of solid tumors. Cofilin-1 is an actin-depolymerizing protein, which is essential for the dynamic changes in the actin cytoskeleton. Cofilin-1 expression increases in relation to cell cycle progression, migration, intravasation and the invasion of cancer cells. Ezrin, which organizes membrane-cytoskeleton-associated complexes, is clearly associated with a poor prognosis and metastasis in different cancer types. Actinin-4 (ACTN4) is highly concentrated in actin-reach protrusions and invadopodias at the peripheries of cell clusters and induces cancer invasion. Actin remodeling in cancer cells may be the result of the inactivation of the actin-binding protein Gelsolin. Fascin is an actin cytoskeletal protein that supports the development of membrane protrusions, stabilizes actin in invadopodia and potentiates protrusive invasion, also serving as a molecular linker between type I receptors and the actin cytoskeleton to facilitate the trafficking of internalized receptors from clathrin-coated vesicles to early endosomes in endocytosis. 

## 6. Protein Phosphorylation 

Protein phosphorylation is a crucial cellular event that is involved in the most important processes of cell migration and invasion. Altered tyrosine phosphorylation signals in cancer cells contribute to a number of aberrant characteristics involved in tumor invasion and metastasis (i.e., focal adhesion assembly, actin cytoskeleton remodeling) (Table 4). Cell motility is stimulated by extracellular stimuli and initiated by intracellular signaling proteins that localize to sites of cell contact with the extracellular matrix, and it is termed focal contacts or focal adhesion. Focal adhesion kinase (FAK) is an intracellular protein-tyrosine kinase that acts to regulate the cycle of the focal contact formation and disassembly required for efficient cell movement. FAK is activated via autophosphorylation at tyrosine 397 (Y397), which is initiated by integrin engagement with its ligand. When phosphorylated, Y397 becomes a binding site for the tyrosine kinase Src, which phosphorylates FAK at Y576 and Y577 to further activate FAK kinase activity [163]. Src also phosphorylates Y861 and Y925, creating docking sites for other SH2 domain-bearing molecules, such as Grb2, which links FAK to activation of Ras and the MAPK pathway [164,165]. FAK phosphorylation via Src causes FAK to be excluded from focal adhesions [166]. Confilin, an actin-binding protein that plays an essential role in regulating acting filament dynamics and reorganization, is inactivated by phosphorylation at the serine residue at position 3. The inactivation of confilin blocks its acting severing and depolymerization activities. There is increasing evidence that confilin phosphorylation is a key link connecting extracellular stimuli to acting cytoskeleton dynamics [167]. MyosinⅡ, as a major motor protein responsible for the generation of cytoskeleton tension, can regulate filament stability, permitting the rapid remodeling of the actomyosin cytoskeleton through phosphorylation [168]. Therefore, phosphorylation is an integral part of tumor cell motility, migration and signaling. 

For phosphoproteomics analysis, TiO2 phosphopeptide enrichment spin tips are usually employed after TMT labeling to increase the signal-to-noise ratio of endogenous phosphopeptide in digested cells. The phosphor-site localization used to be determined by Ascore approach [179]. The proteome combined with phosphoproteome will depict the complete molecular signatures of the highly invasive subpopulations, and the comparison with original cell lines will identify the special markers only expressed on highly invasive subpopulations.

## 7. Conclusions

Undoubtedly, cell mechanotype provides information about the invasive propensity of cancer cells and is thus emerging as a complementary biomarker for malignancy. It is still unclear whether the increased metastatic cell deformability drives invasive potential, or whether the selective pressures applied during metastatic progression induce deformability changes. Nevertheless, mechanobiology-based markers may be potentially important for cancer treatment and outcomes. We hope that our concise review will contribute to the emergence of a comprehensive and appropriate set of clinically significant proteomic-based biomarkers of various mechanotypes in the near future. 

The creation of new technologies for predicting and treating cancer metastasis, which is the main cause of cancer-related death, will serve to form scientific and technological groundwork on the basis of inter- and multidisciplinary approaches. In addition, it will contribute to the identification of factors that are responsible for homeostasis-metabolism failure and affect human cell function and lifespan. Understanding the molecular pathways that contribute to the cancer-related deterioration of cell function and developing methods to modulate them will be critical for the development of therapeutic interventions to promote healthy longevity on a patient-specific basis. Moreover, the identification of biomarkers reflecting the degree of cancer, and most importantly, the treatment success, will increase their potential use in clinical applications. These studies may therefore have a direct and powerful impact on public health initiatives and research funding with potentially wide-reaching effects.

## Figures and Tables

**Table 1:** The similarities in intracellular process and compartments during endocytosis and invasion/migration process by cancer cells.

Intracellular Process/Compartment	Invasion	Endocytosis	Reference
**Actin polymerization**	Actin polymerization occurs in cells at the leading edge of the invadosome during invasion	Actin polymerization promotes the movement of the nascent endocytic vesicles into the cytoplasmic milieu, forming a comet tail	[82,83]
**Myosin**	Myosin phosphorylation by Cdc42-MRCK and Rho-ROCK signaling coordinates cell invasion	Myosin coordinates actin assembly and cargo trafficking during clathrin-mediated endocytosis	[84,85]
**Formation of stress fibers**	RhoA and RhoC are major GTpases capable of mediating stress fiber formation and generating the contractile force needed for retraction of the trailing edge during migration and invasion	The endocytotic protein Caveolin-1 regulates tension from stress fibers via RhoA signaling	[86,87]
**EMT**	Loss of E-cadherin is leading to acquisition of migration characteristics through loss of adhesive junctions	E-cadherin internalization is mediated by clathrin-mediated endocytosis, caveolin-mediated endocytosis, and macropinocytosis	[88,89]
**Integrin trafficking**	It promotes invasion, Rab 25 gene delivers α5b1, an integrin influences invasion to pseudopod tips at the plasma membrane	Integrins trafficking is driven by Rab which mediates clathrin-dependent and -independent endocytosis.	[90,91,92,93,94]
**Integrin recycling**	Integrin recycling is coordinated by Rab-coupling protein pathway and RTK, this drives invasion into fibronectin-rich 3D ECM.	The integrin is recycled through Rab11- and/or Arf6-dependent mechanisms in the endosomal system.	[95,96]
**Cell adhesion**	Many adhesion and signaling molecules are involved in cell migration and tumor invasion, including integrins, CD44, and several (IgCAMs).	CD44 mediates the endocytosis	[97,98,99,100]
**Catenin signaling**	The Wnt/β-catenin signaling pathway is a receptor-dependent mechanism coordinated by the Fzd receptor to facilitate cell invasion.	Β-catenin-dependent Wnt ligands require endocytosis for signal activation and to regulate gene transcription in the responding cells	[101,102]
**Microtubule network**	Microtubules help to form and maintain membrane protrusions by their ability to withstand high compressive loads and generate pushing forces employed by migrating cells.	Microtubules can transport recycling endosomes containing membrane-associated signaling molecules which are required for cell migration.	[103,104]

**Table 2:** Signaling pathways for cancer cell invasion/migration and potentially clinically relevant biomarkers.

Signalling Pathway	Function during Cell Invasion and/or Migration	Potential Clinically Relevant Biomarker	Reference
**EGF**	Promotes epithelial-mesenchymal transition (EMT) through the B-catenin stabilization	ECM1	[125,126]
**Ras**	Participates in membrane and cytoskeletal remodeling during endocytic transport	Annexin A6	[127]
**FAK/PI3K**	Participates in the phenotype changes in focal adhesion and cytoskeletal dynamics and alteration in the activation of MMPs	Galectin-1, ITGA5	[128,129]
**ERK**	Dissembles adhesion process to facilitate lamellipodium protrusion	GIT1	[130,131]
**WAVE**	Helps actin polymerization for the formation of lamellipodia and amoeboid movement	WAVE2	[132,133]
**Wnt/β-catenin**	Allows cytoskeleton reorganization by the activation of small GTpases Rho or Rac, triggering ROCK downstream	Mucin 1	[102,134,135]
**FAK**	Participates in cell migration, activation of Rho-GTpases, integrin signalling	Rho GTPase, MMPs, CXCR1	[136,137,138]
**Hippo**	Promotes the downregulation of EMT machinery (E-cadherin and Laminin), cell proliferation and apoptosis, tumorigenesis	Yap-1, YAP-TAZ	[139,140,141,142]
**Notch**	Induces EMT via the activation of transcriptional repressor proteins leading to E-cadherin downregulation	Notch 3	[143,144]
**P53**	Regulates DNA repair, control of the cell cycle, apoptosis, and differentiation	p53 protein	[145,146]

**Table 3:** Ten potential marker candidates that are directly related to actin cytoskeleton remodeling in cancer cells.

Marker	Cancer Type	Involvement	Reference
**Annexin A6 and A2**	Melanoma, cervical cancer, epithelial carcinoma, breast cancer, gastric cancer, prostate cancer, acute lymphoblastic leukemia, chronic myeloid leukemia, large-cell lymphoma myeloma	Formation of reversible, membrane-cytoskeleton complexes through interactions with actin and α-actinin	[147,148]
**ECM1**	Epithelial tumors, invasive breast cancer	Regulates cell proliferation, enhances MUC1 expression and stabilizes EGFR/HER3 proteins via a galectin-3/MUC1-dependent mechanism stabilization of β-catenin	[125,149]
**Transgelin**	Colorectal cancer	Stabilization of actin filaments promotes actin gelling is involved in podosome formation in smooth muscle cells, thus predisposing the cells toward migration and invasion. It is associated with Ca2+-independent vascular contractility and is also a direct target of transforming growth factor β (TGF-β)/Smad3-dependent epithelial cell migration in idiopathic pulmonary fibrosis	[150,151,152]
**PROF1**	Human colon cancer	Actin-monomer binding protein regulates actin dynamics and cell motility and plays an important role in the migration of cancer cells	[153]
**Myosin 9**	Lung cancer, breast cancer, leukemia, gastric cancer, esophageal cancer, and other malignant tumors	A cytokine, involved in cytoskeletal reorganization; plays an important role in the formation of cellular pseudopodia	[154]
**Cofilin-1**	Colorectal cancer	Actin-depolymerizing protein increases in relation to cell cycle progression, migration, intravasation and the invasion	[155]
**Ezrin**	Osteosarcoma, pancreatic cancer, lung cancer, and others	Actin filament binding proteinfacilitates numerous signal transductions in tumorigenesis and mediates diverse essential functions through interactions with a variety of growth factor receptors and adhesion molecules	[156]
** ACTN4 **	Carcinoma tongue cancer, pancreatic cancer, lung cancers	Highly concentrated in actin-rich protrusions and invadopodias at the peripheries of cell clusters, induces cancer invasion	[157]
**Gelsolin**	Hepatocellular carcinoma	A cytoskeletal protein, frequently overexpressed in different cancers and promotes cell motility	[158]
**Fascin**	Breast cancer	A promoter of directed cell migration supporting the development of membrane protrusions	[159,160,161,162]

**Table 4:** Functions of specific phosphorylation sites.

Protein	Phosphorylation Site	Function	Reference
**FAK**	Y397,	Autophosphorylation; binding site for Src family kinase (SFKs), p85	[165,169,170,171]
	Y576, Y577	Regulate the catalytic activity	[172]
	Y861, Y925	Serve as a docking site for SH2 domain-containing proteins	[165,173,174]
**Cofilin**	Ser3	Inhibit its binding to G-actin and F-actin, inactivate itself	[175,176]
**Myosin** **Ⅱ**	Ser19, Thr18	Increase the Mg^2+^-ATPase activity of myosin	[168,177]
	Ser1943	Regulates the motility of breast cancer cells	[178]

## Data Availability

Not applicable.

## References

[1] Yang J., Weinberg R.A. (2008). Epithelial-Mesenchymal Transition: At the Crossroads of Development and Tumor Metastasis. Dev. Cell.

[2] Sleeman J.P., Nazarenko I., Thiele W. (2011). Do All Roads Lead to Rome? Routes to Metastasis Development. Int. J. Cancer.

[3] Cairns R.A., Khokha R., Hill R.P. (2003). Molecular Mechanisms of Tumor Invasion and Metastasis: An Integrated View. Curr. Mol. Med..

[4] Weigelt B., Peterse J.L., van ’t Veer L.J. (2005). Breast Cancer Metastasis: Markers and Models. Nat. Rev. Cancer.

[5] Riihimaki M., Thomsen H., Hemminki A., Sundquist K., Hemminki K., Riihimäki M., Thomsen H., Hemminki A., Sundquist K., Hemminki K. (2013). Comparison of Survival of Patients with Metastases from Known versus Unknown Primaries: Survival in Metastatic Cancer. BMC Cancer.

[6] Lynch J.A., Venne V., Berse B. (2015). Genetic Tests to Identify Risk for Breast Cancer. Semin. Oncol. Nurs..

[7] Vaidyanathan K., Vasudevan D.M. (2012). Organ Specific Tumor Markers: What’s New?. Indian J Clin Biochem.

[8] Gurcan M.N., Boucheron L.E., Can A., Madabhushi A., Rajpoot N.M., Yener B. (2009). Histopathological Image Analysis: A Review. IEEE Rev. Biomed. Eng..

[9] Wei J.W., Tafe L.J., Linnik Y.A., Vaickus L.J., Tomita N., Hassanpour S. (2019). Pathologist-Level Classification of Histologic Patterns on Resected Lung Adenocarcinoma Slides with Deep Neural Networks. Sci. Rep..

[10] Lang E.V., Berbaum K.S., Lutgendorf S.K. (2009). Large-Core Breast Biopsy: Abnormal Salivary Cortisol Profiles Associated with Uncertainty of Diagnosis. Radiology.

[11] Yankaskas C.L., Thompson K.N., Paul C.D., Vitolo M.I., Mistriotis P., Mahendra A., Bajpai V.K., Shea D.J., Manto K.M., Chai A.C. (2019). A Microfluidic Assay for the Quantification of the Metastatic Propensity of Breast Cancer Specimens. Nat. Biomed. Eng..

[12] Murugan A.K. (2019). MTOR: Role in Cancer, Metastasis and Drug Resistance. Semin. Cancer Biol..

[13] Wu L., Qu X. (2015). Cancer Biomarker Detection: Recent Achievements and Challenges. Chem. Soc. Rev..

[14] Narayan Bhatt A., Farooque A., Verma A. (2010). Cancer Biomarkers-Current Perspectives Role of Tumor Microenvironment in Treatment of Lymphoma and Myeloma View Project Metabolic Signaling Approaches for Anticancer Drug Target Discovery View Project. Artic. Indian J. Med. Res..

[15] Wang F., Fang Q., Ge Z., Yu N., Xu S., Fan X. (2012). Common BRCA1 and BRCA2 Mutations in Breast Cancer Families: A Meta-Analysis from Systematic Review. Mol. Biol. Rep..

[16] De P., Mukhopadhyay M.J. (2021). Study of the Chromosomal Abnormalities and Associated Complex Karyotypes in Hematological Cancer in the Population of West Bengal: A Prospective Observational Study. Indian J. Med. Paediatr. Oncol..

[17] Wang C., Bai F., Zhang L.Z., Scott A., Li E., Pei X.H. (2018). Estrogen Promotes Estrogen Receptor Negative BRCA1-Deficient Tumor Initiation and Progression. Breast Cancer Res..

[18] Huang J., Duran A., Reina-Campos M., Valencia T., Castilla E.A., Müller T.D., Tschöp M.H., Moscat J., Diaz-Meco M.T. (2018). Adipocyte P62/SQSTM1 Suppresses Tumorigenesis through Opposite Regulations of Metabolism in Adipose Tissue and Tumor. Cancer Cell.

[19] Szablewski L. (2022). Glucose Transporters as Markers of Diagnosis and Prognosis in Cancer Diseases. Oncol. Rev..

[20] Pouliquen D., Boissard A., Coqueret O., Guette C. (2020). Biomarkers of Tumor Invasiveness in Proteomics (Review). Int. J. Oncol..

[21] Wilt T.J., Scardino P.T., Carlsson S.V., Basch E. (2014). Prostate-Specific Antigen Screening in Prostate Cancer: Perspectives on the Evidence. J. Natl. Cancer Inst..

[22] Brufsky A.M., Mayer M., Rugo H.S., Kaufman P.A., Tan-Chiu E., Tripathy D., Tudor I.C., Wang L.I., Brammer M.G., Shing M. (2011). Central Nervous System Metastases in Patients with HER2-Positive Metastatic Breast Cancer: Incidence, Treatment, and Survival in Patients from RegistHER. Clin. Cancer Res..

[23] Kuba S., Ishida M., Nakamura Y., Yamanouchi K., Minami S., Taguchi K., Eguchi S., Ohno S. (2014). Treatment and Prognosis of Breast Cancer Patients with Brain Metastases According to Intrinsic Subtype. Jpn. J. Clin. Oncol..

[24] Molinie N., Rubtsova S.N., Fokin A., Visweshwaran S.P., Rocques N., Polesskaya A., Schnitzler A., Vacher S., Denisov E.V., Tashireva L.A. (2019). Cortical Branched Actin Determines Cell Cycle Progression. Cell Res..

[25] Geiger T., Cox J., Mann M. (2010). Proteomic Changes Resulting from Gene Copy Number Variations in Cancer Cells. PLoS Genet..

[26] Murakami T., Kikuchi H., Ishimatsu H., Iino I., Hirotsu A., Matsumoto T., Ozaki Y., Kawabata T., Hiramatsu Y., Ohta M. (2017). Tenascin C in Colorectal Cancer Stroma Is a Predictive Marker for Liver Metastasis and Is a Potent Target of MiR-198 as Identified by MicroRNA Analysis. Br. J. Cancer.

[27] Guck J., Schinkinger S., Lincoln B., Wottawah F., Ebert S., Romeyke M., Lenz D., Erickson H.M., Ananthakrishnan R., Mitchell D. (2005). Optical Deformability as an Inherent Cell Marker for Testing Malignant Transformation and Metastatic Competence. Biophys. J..

[28] Qi D., Gill N.K., Santiskulvong C., Sifuentes J., Dorigo O., Rao J., Taylor-Harding B., Wiedemeyer W.R., Rowat A.C. (2015). Screening Cell Mechanotype by Parallel Microfiltration. Sci. Rep..

[29] Byun S., Son S., Amodei D., Cermak N., Shaw J., Kang J.H., Hecht V.C., Winslow M.M., Jacks T., Mallick P. (2013). Characterizing Deformability and Surface Friction of Cancer Cells. Proc. Natl. Acad. Sci. USA.

[30] Paszek M.J., Zahir N., Johnson K.R., Lakins J.N., Rozenberg G.I., Gefen A., Reinhart-King C.A., Margulies S.S., Dembo M., Boettiger D. (2005). Tensional Homeostasis and the Malignant Phenotype. Cancer Cell.

[31] Guck J., Ananthakrishnan R., Mahmood H., Moon T.J., Cunningham C.C., Käs J. (2001). The Optical Stretcher: A Novel Laser Tool to Micromanipulate Cells. Biophys. J..

[32] Gossett D.R., Tse H.T.K., Lee S.A., Ying Y., Lindgren A.G., Yang O.O., Rao J., Clark A.T., Di Carlo D. (2012). Hydrodynamic Stretching of Single Cells for Large Population Mechanical Phenotyping. Proc. Natl. Acad. Sci. USA.

[33] Lekka M., Laidler P., Gil D., Lekki J., Stachura Z., Hrynkiewicz A.Z. (1999). Elasticity of Normal and Cancerous Human Bladder Cells Studied by Scanning Force Microscopy. Eur. Biophys. J..

[34] Mak M., Spill F., Kamm R.D., Zaman M.H. (2016). Single-Cell Migration in Complex Microenvironments: Mechanics and Signaling Dynamics. J. Biomech. Eng..

[35] Pachenari M., Seyedpour S.M., Janmaleki M., Shayan S.B., Taranejoo S., Hosseinkhani H. (2014). Mechanical Properties of Cancer Cytoskeleton Depend on Actin Filaments to Microtubules Content: Investigating Different Grades of Colon Cancer Cell Lines. J. Biomech..

[36] Ketene A.N., Schmelz E.M., Roberts P.C., Agah M. (2012). The Effects of Cancer Progression on the Viscoelasticity of Ovarian Cell Cytoskeleton Structures. Nanomedicine.

[37] Ochalek T., Nordt F.J., Tullberg K., Burger M.M. (1988). Correlation between Cell Deformability and Metastatic Potential in B16-F1 Melanoma Cell Variants. Cancer Res..

[38] Kramer N., Walzl A., Unger C., Rosner M., Krupitza G., Hengstschläger M., Dolznig H. (2013). In Vitro Cell Migration and Invasion Assays. Mutat. Res.—Rev. Mutat. Res..

[39] Paul C.D., Mistriotis P., Konstantopoulos K. (2017). Cancer Cell Motility: Lessons from Migration in Confined Spaces. Nat. Rev. Cancer.

[40] Van Golen K.L., Wu Z.F., Xiao X.T., Bao L.W., Merajver S.D. (2000). RhoC GTPase, a Novel Transforming Oncogene for Human Mammary Epithelial Cells That Partially Recapitulates the Inflammatory Breast Cancer Phenotype. Cancer Res..

[41] Lin M., DiVito M.M., Merajver S.D., Boyanapalli M., van Golen K.L. (2005). Regulation of Pancreatic Cancer Cell Migration and Invasion by RhoC GTPase and Caveolin-1. Mol. Cancer.

[42] Shelby J.P., White J., Ganesan K., Rathod P.K., Chiu D.T. (2003). A Microfluidic Model for Single-Cell Capillary Obstruction by Plasmodium Falciparum-Infected Erythrocytes. Proc. Natl. Acad. Sci. USA.

[43] Lautscham L.A., Kämmerer C., Lange J.R., Kolb T., Mark C., Schilling A., Strissel P.L., Strick R., Gluth C., Rowat A.C. (2015). Migration in Confined 3D Environments Is Determined by a Combination of Adhesiveness, Nuclear Volume, Contractility, and Cell Stiffness. Biophys. J..

[44] Mak M., Reinhart-King C.A., Erickson D. (2013). Elucidating Mechanical Transition Effects of Invading Cancer Cells with a Subnucleus-Scaled Microfluidic Serial Dimensional Modulation Device. Lab Chip.

[45] Albini A., Benelli R. (2007). The Chemoinvasion Assay: A Method to Assess Tumor and Endothelial Cell Invasion and Its Modulation. Nat. Protoc..

[46] McEwan R.N., Kleinman H.K., Martin G.R. (1987). A Rapid in Vitro Assay for Quantitating the Invasive Potential of Tumor Cells. Cancer Res..

[47] Justus C.R., Leffler N., Ruiz-Echevarria M., Yang L.V. (2014). In Vitro Cell Migration and Invasion Assays. J. Vis. Exp..

[48] Sieuwerts A.M., Klijn J.G.M., Foekens J.A. (1997). Assessment of the Invasive Potential of Human Gynecological Tumor Cell Lines with the in Vitro Boyden Chamber Assay: Influences of the Ability of Cells to Migrate through the Filter Membrane. Clin. Exp. Metastasis.

[49] Nyström M.L., Thomas G.J., Stone M., Mackenzie I.C., Hart I.R., Marshall J.F. (2005). Development of a Quantitative Method to Analyse Tumour Cell Invasion in Organotypic Culture. J. Pathol..

[50] Ayala I., Baldassarre M., Caldieri G., Buccione R. (2006). Invadopodia: A Guided Tour. Eur. J. Cell Biol..

[51] Artym V.V., Yamada K.M., Mueller S.C. (2009). ECM Degradation Assays for Analyzing Local Cell Invasion. Methods Mol. Biol..

[52] Merkher Y., Horesh Y., Abramov Z., Shleifer G., Ben-Ishay O., Kluger Y., Weihs D. (2020). Rapid Cancer Diagnosis and Early Prognosis of Metastatic Risk Based on Mechanical Invasiveness of Sampled Cells. Ann. Biomed. Eng..

[53] Merkher Y., Weihs D. (2017). Proximity of Metastatic Cells Enhances Their Mechanobiological Invasiveness. Ann. Biomed. Eng..

[54] Merkher Y., Alvarez-Elizondo M.B., Weihs D. (2017). Taxol Reduces Synergistic, Mechanobiological Invasiveness of Metastatic Cells. Converg. Sci. Phys. Oncol..

[55] Merkher Y., Kontareva E., Melekhova A., Leonov S. (2021). Abstract PO-042: Nanoparticles Imaging for Cancer Metastasis Diagnosis. Clin. Cancer Res..

[56] (2021). Merkher Yulia; Kontareva Elizaveta; Bogdan Elizaveta; Achkasov Konstantin; Grolman Joshua; Leonov Sergey Nanoparticle Cellular Endocytosis as Potential Prognostic Biomarker for Cancer Progression. FEBS Open Bio.

[57] Li Y., Zhang H., Merkher Y., Chen L., Liu N., Leonov S., Chen Y. (2022). Recent Advances in Therapeutic Strategies for Triple-Negative Breast Cancer. J. Hematol. Oncol..

[58] Ghosh M., Song X., Mouneimne G., Sidani M., Lawrence D.S., Condeelis J.S. (2004). Cofilin Promotes Actin Polymerization and Defines the Direction of Cell Motility. Science.

[59] Ballestrem C., Wehrle-Haller B., Hinz B., Imhof B.A. (2000). Actin-Dependent Lamellipodia Formation and Microtubule-Dependent Tail Retraction Control-Directed Cell Migration. Mol. Biol. Cell.

[60] Campellone K.G., Welch M.D. (2010). A Nucleator Arms Race: Cellular Control of Actin Assembly. Nat. Rev. Mol. Cell Biol..

[61] Yang Q., Zhang X.F., Pollard T.D., Forscher P. (2012). Arp2/3 Complex-Dependent Actin Networks Constrain Myosin II Function in Driving Retrograde Actin Flow. J. Cell Biol..

[62] Génot E., Gligorijevic B. (2014). Invadosomes in Their Natural Habitat. Eur. J. Cell Biol..

[63] Winograd-Katz S.E., Brunner M.C., Mirlas N., Geiger B. (2011). Analysis of the Signaling Pathways Regulating Src-Dependent Remodeling of the Actin Cytoskeleton. Eur. J. Cell Biol..

[64] Destaing O., Block M.R., Planus E., Albiges-Rizo C. (2011). Invadosome Regulation by Adhesion Signaling. Curr. Opin. Cell Biol..

[65] Schmidt S., Nakchbandi I., Ruppert R., Kawelke N., Hess M.W., Pfaller K., Jurdic P., Fässler R., Moser M. (2011). Kindlin-3-Mediated Signaling from Multiple Integrin Classes Is Required for Osteoclast-Mediated Bone Resorption. J. Cell Biol..

[66] Mierke C.T. (2020). Mechanical Cues Affect Migration and Invasion of Cells From Three Different Directions. Front. Cell Dev. Biol..

[67] Akamatsu M., Vasan R., Serwas D., Ferrin M., Rangamani P., Drubin D.G. (2020). Principles of Self-Organization and Load Adaptation by the Actin Cytoskeleton during Clathrin-Mediated Endocytosis. Elife.

[68] Weston L., Coutts A.S., La Thangué N.B. (2012). Actin Nucleators in the Nucleus: An Emerging Theme. J. Cell Sci..

[69] Pollard T.D., Cooper J.A. (2009). Actin, a Central Player in Cell Shape and Movement. Science.

[70] Sirotkin V. (2011). Cell Biology: Actin Keeps Endocytosis on a Short Leash. Curr. Biol..

[71] Gheorghe D.M., Aghamohammadzadeh S., Smaczynska-de Rooij I.I., Allwood E.G., Winder S.J., Ayscough K.R. (2008). Interactions between the Yeast SM22 Homologue Scp1 and Actin Demonstrate the Importance of Actin Bundling in Endocytosis. J. Biol. Chem..

[72] Kim K., Galletta B.J., Schmidt K.O., Chang F.S., Blumer K.J., Cooper J.A. (2006). Actin-Based Motility during Endocytosis in Budding Yeast. Mol. Biol. Cell.

[73] Kaksonen M., Toret C.P., Drubin D.G. (2005). A Modular Design for the Clathrin- and Actin-Mediated Endocytosis Machinery. Cell.

[74] Galletta B.J., Cooper J.A. (2009). Actin and Endocytosis: Mechanisms and Phylogeny. Curr. Opin. Cell Biol..

[75] Pishvaee B., Costaguta G., Yeung B.G., Ryazantsev S., Greener T., Greene L.E., Eisenberg E., McCaffery J.M., Payne G.S. (2000). A Yeast DNA J Protein Required for Uncoating of Clathrin-Coated Vesicles in Vivo. Nat. Cell Biol..

[76] Toret C.P., Lee L., Sekiya-Kawasaki M., Drubin D.G. (2008). Multiple Pathways Regulate Endocytic Coat Disassembly in Saccharomyces Cerevisiae for Optimal Downstream Trafficking. Traffic.

[77] Galletta B.J., Chuang D.Y., Cooper J.A. (2008). Distinct Roles for Arp2/3 Regulators in Actin Assembly and Endocytosis. PLoS Biol..

[78] Merrifield C.J., Qualmann B., Kessels M.M., Almers W. (2004). Neural Wiskott Aldrich Syndrome Protein (N-WASP) and the Arp 2/3 Complex Are Recruited to Sites of Clathrin-Mediated Endocytosis in Cultured Fibroblasts. Eur. J. Cell Biol..

[79] Jonas O., Mierke C.T., Käs J.A. (2011). Invasive Cancer Cell Lines Exhibit Biomechanical Properties That Are Distinct from Their Noninvasive Counterparts. Soft Matter.

[80] Smythe E., Ayscough K.R. (2006). Actin Regulation in Endocytosis. J. Cell Sci..

[81] Alvarez-Elizondo M.B.M.B.M.B., Merkher Y., Shleifer G., Gashri C., Weihs D. (2020). Actin as a Target to Reduce Cell Invasiveness in Initial Stages of Metastasis. Ann. Biomed. Eng..

[82] Bearer E.L. (1993). Role of Actin Polymerization in Cell Locomotion: Molecules and Models. Am. J. Respir. Cell Mol. Biol..

[83] Taunton J., Rowning B.A., Coughlin M.L., Wu M., Moon R.T., Mitchison T.J., Larabell C.A. (2000). Actin-Dependent Propulsion of Endosomes and Lysosomes by Recruitment of N-WASP. J. Cell Biol..

[84] Cheng J., Grassart A., Drubin D.G. (2012). Myosin 1E Coordinates Actin Assembly and Cargo Trafficking during Clathrin-Mediated Endocytosis. Mol. Biol. Cell.

[85] Wilkinson S., Paterson H.F., Marshall C.J. (2005). Cdc42-MRCK and Rho-ROCK Signalling Cooperate in Myosin Phosphorylation and Cell Invasion. Nat. Cell Biol..

[86] Echarri A., Del Pozo M.A. (2015). Caveolae—Mechanosensitive Membrane Invaginations Linked to Actin Filaments. J. Cell Sci..

[87] O’Connor K.L., Chen M. (2013). Dynamic Functions of RhoA in Tumor Cell Migration and Invasion. Small GTPases.

[88] Cavallaro U., Christofori G. (2004). Cell Adhesion and Signalling by Cadherins and Ig-CAMs in Cancer. Nat. Rev. Cancer.

[89] Shankar J., Nabi I.R. (2015). Actin Cytoskeleton Regulation of Epithelial Mesenchymal Transition in Metastatic Cancer Cells. PLoS ONE.

[90] Bor L.T., Ee L.N. (2009). Rabs and Cancer Cell Motility. Cell Motil. Cytoskelet..

[91] Caswell P.T., Spence H.J., Parsons M., White D.P., Clark K., Cheng K.W., Mills G.B., Humphries M.J., Messent A.J., Anderson K.I. (2007). Rab25 Associates with A5β1 Integrin to Promote Invasive Migration in 3D Microenvironments. Dev. Cell.

[92] Cheng K.W., Lahad J.P., Kuo W.L., Lapuk A., Yamada K., Auersperg N., Liu J., Smith-McCune K., Lu K.H., Fishman D. (2004). The RAB25 Small GTPase Determines Aggressiveness of Ovarian and Breast Cancers. Nat. Med..

[93] Jeong B.Y., Cho K.H., Jeong K.J., Park Y.Y., Kim J.M., Rha S.Y., Park C.G., Mills G.B., Cheong J.H., Lee H.Y. (2018). Rab25 Augments Cancer Cell Invasiveness through a Β1 Integrin/EGFR/VEGF-A/Snail Signaling Axis and Expression of Fascin. Exp. Mol. Med..

[94] Paul N.R., Jacquemet G., Caswell P.T. (2015). Endocytic Trafficking of Integrins in Cell Migration. Curr. Biol..

[95] Caswell P.T., Chan M., Lindsay A.J., McCaffrey M.W., Boettiger D., Norman J.C. (2008). Rab-Coupling Protein Coordinates Recycling of A5β1 Integrin and EGFR1 to Promote Cell Migration in 3D Microenvironments. J. Cell Biol..

[96] Jones M.C., Caswell P.T., Norman J.C. (2006). Endocytic Recycling Pathways: Emerging Regulators of Cell Migration. Curr. Opin. Cell Biol..

[97] Kawauchi T. (2012). Cell Adhesion and Its Endocytic Regulation in Cell Migration during Neural Development and Cancer Metastasis. Int. J. Mol. Sci..

[98] Weber G.F. (2008). Molecular Mechanisms of Metastasis. Cancer Lett..

[99] Ponta H., Sherman L., Herrlich P.A. (2003). CD44: From Adhesion Molecules to Signalling Regulators. Nat. Rev. Mol. Cell Biol..

[100] Müller S., Sindikubwabo F., Cañeque T., Lafon A., Versini A., Lombard B., Loew D., Wu T., Wu T.D., Ginestier C. (2020). CD44 Regulates Epigenetic Plasticity by Mediating Iron Endocytosis. Nat. Chem..

[101] Rim E.Y., Kinney L.K., Nusse R. (2020). β-Catenin-Mediated Wnt Signal Transduction Proceeds through an Endocytosis-Independent Mechanism. Mol. Biol. Cell.

[102] Zhu W., Wang H., Zhu D. (2022). Wnt/β-Catenin Signaling Pathway in Lung Cancer. Med. Drug Discov..

[103] Garcin C., Straube A. (2019). Microtubules in Cell Migration. Essays Biochem..

[104] Palamidessi A., Frittoli E., Garré M., Faretta M., Mione M., Testa I., Diaspro A., Lanzetti L., Scita G., Di Fiore P.P. (2008). Endocytic Trafficking of Rac Is Required for the Spatial Restriction of Signaling in Cell Migration. Cell.

[105] Yamaguchi H., Condeelis J. (2007). Regulation of the Actin Cytoskeleton in Cancer Cell Migration and Invasion. Biochim. Biophys. Acta—Mol. Cell Res..

[106] Iwaya K., Norio K., Mukai K. (2007). Coexpression of Arp2 and WAVE2 Predicts Poor Outcome in Invasive Breast Carcinoma. Mod. Pathol..

[107] Sharma M., Sah P., Sharma S., Radhakrishnan R. (2013). Molecular Changes in Invasive Front of Oral Cancer. J. Oral Maxillofac. Pathol..

[108] Clark E.S., Brown B., Whigham A.S., Kochaishvili A., Yarbrough W.G., Weaver A.M. (2009). Aggressiveness of HNSCC Tumors Depends on Expression Levels of Cortactin, a Gene in the 11q13 Amplicon. Oncogene.

[109] Weaver A.M., Karginov A.V., Kinley A.W., Weed S.A., Li Y., Parsons J.T., Cooper J.A. (2001). Cortactin Promotes and Stabilizes Arp2/3-Induced Actin Filament Network Formation. Curr. Biol..

[110] Mader C.C., Oser M., Magalhaes M.A.O., Bravo-Cordero J.J., Condeelis J., Koleske A.J., Gil-Henn H. (2011). An EGFR-Src-Arg-Cortactin Pathway Mediates Functional Maturation of Invadopodia and Breast Cancer Cell Invasion. Cancer Res..

[111] Zhu J., Yu D., Zeng X.C., Zhou K., Zhan X. (2007). Receptor-Mediated Endocytosis Involves Tyrosine Phosphorylation of Cortactin. J. Biol. Chem..

[112] Trochet D., Bitoun M. (2021). A Review of Dynamin 2 Involvement in Cancers Highlights a Promising Therapeutic Target. J. Exp. Clin. Cancer Res..

[113] Molinie N., Gautreau A. (2018). The Arp2/3 Regulatory System and Its Deregulation in Cancer. Physiol. Rev..

[114] Papalazarou V., Machesky L.M. (2021). The Cell Pushes Back: The Arp2/3 Complex Is a Key Orchestrator of Cellular Responses to Environmental Forces. Curr. Opin. Cell Biol..

[115] Gerashchenko T.S., Novikov N.M., Krakhmal N.V., Zolotaryova S.Y., Zavyalova M.V., Cherdyntseva N.V., Denisov E.V., Perelmuter V.M. (2019). Markers of Cancer Cell Invasion: Are They Good Enough?. J. Clin. Med..

[116] Jiang X., Xie H., Dou Y., Yuan J., Zeng D., Xiao S. (2020). Expression and Function of FRA1 Protein in Tumors. Mol. Biol. Rep..

[117] Llorens M.C., Rossi F.A., García I.A., Cooke M., Abba M.C., Lopez-Haber C., Barrio-Real L., Vaglienti M.V., Rossi M., Bocco J.L. (2019). PKCα Modulates Epithelial-to-Mesenchymal Transition and Invasiveness of Breast Cancer Cells Through ZEB1. Front. Oncol..

[118] Vuoriluoto K., Haugen H., Kiviluoto S., Mpindi J.P., Nevo J., Gjerdrum C., Tiron C., Lorens J.B., Ivaska J. (2011). Vimentin Regulates EMT Induction by Slug and Oncogenic H-Ras and Migration by Governing Axl Expression in Breast Cancer. Oncogene.

[119] Geletu M., Mohan R., Arulanandam R., Berger-Becvar A., Nabi I.R., Gunning P.T., Raptis L. (2018). Reciprocal Regulation of the Cadherin-11/Stat3 Axis by Caveolin-1 in Mouse Fibroblasts and Lung Carcinoma Cells. Biochim. Biophys. Acta—Mol. Cell Res..

[120] Arpaia E., Blaser H., Quintela-Fandino M., Duncan G., Leong H.S., Ablack A., Nambiar S.C., Lind E.F., Silvester J., Fleming C.K. (2012). The Interaction between Caveolin-1 and Rho-GTPases Promotes Metastasis by Controlling the Expression of Alpha5-Integrin and the Activation of Src, Ras and Erk. Oncogene.

[121] Ni K., Wang C., Carnino J.M., Jin Y. (2020). The Evolving Role of Caveolin-1: A Critical Regulator of Extracellular Vesicles. Med. Sci..

[122] Henry N.L., Hayes D.F. (2012). Cancer Biomarkers. Mol. Oncol..

[123] Goicoechea S.M., Bednarski B., García-Mata R., Prentice-Dunn H., Kim H.J., Otey C.A. (2009). Palladin Contributes to Invasive Motility in Human Breast Cancer Cells. Oncogene.

[124] Moreno-Layseca P., Jäntti N.Z., Godbole R., Sommer C., Jacquemet G., Al-Akhrass H., Conway J.R.W., Kronqvist P., Kallionpää R.E., Oliveira-Ferrer L. (2021). Cargo-Specific Recruitment in Clathrin- and Dynamin-Independent Endocytosis. Nat. Cell Biol..

[125] Lee K.-M., Nam K., Oh S., Lim J., Kim Y.P., Lee W.W., Yu J.H., Ahn S.H., Kim S.B., Noh D.Y. (2014). Extracellular Matrix Protein 1 Regulates Cell Proliferation and Trastuzumab Resistance through Activation of Epidermal Growth Factor Signaling. Breast Cancer Res..

[126] Huh Y.H., Oh S., Yeo Y.R., Chae I.H., Kim S.H., Lee J.S., Yun S.J., Choi K.Y., Ryu J.H., Jun C.D. (2015). Swiprosin-1 Stimulates Cancer Invasion and Metastasis by Increasing the Rho Family of GTPase Signaling. Oncotarget.

[127] Qi H., Liu S., Guo C., Wang J., Greenaway F.T., Sun M.Z. (2015). Role of Annexin A6 in Cancer. Oncol. Lett..

[128] Su Y.L., Luo H.L., Huang C.C., Liu T.T., Huang E.Y., Sung M.T., Lin J.J., Chiang P.H., Chen Y.T., Kang C.H. (2020). Galectin-1 Overexpression Activates the FAK/PI3K/AKT/MTOR Pathway and Is Correlated with Upper Urinary Urothelial Carcinoma Progression and Survival. Cells.

[129] Wang J.F., Chen Y.Y., Zhang S.W., Zhao K., Qiu Y., Wang Y., Wang J.C., Yu Z., Li B.P., Wang Z. (2022). ITGA5 Promotes Tumor Progression through the Activation of the FAK/AKT Signaling Pathway in Human Gastric Cancer. Oxid. Med. Cell. Longev..

[130] Chen J., Yang P., Yang J., Wen Z., Zhang B., Zheng X. (2015). GIT1 Is a Novel Prognostic Biomarker and Facilitates Tumor Progression via Activating ERK/MMP9 Signaling in Hepatocellular Carcinoma. OncoTargets Ther..

[131] Mendoza M.C., Vilela M., Juarez J.E., Blenis J., Danuser G. (2015). ERK Reinforces Actin Polymerization to Power Persistent Edge Protrusion during Motility. Sci. Signal..

[132] Taniuchi K., Yawata T., Tsuboi M., Ueba T., Saibara T. (2019). Efficient Delivery of Small Interfering RNAs Targeting Particular MRNAs into Pancreatic Cancer Cells Inhibits Invasiveness and Metastasis of Pancreatic Tumors. Oncotarget.

[133] Rana P.S., Alkrekshi A., Wang W., Markovic V., Sossey-Alaoui K. (2021). The Role of WAVE2 Signaling in Cancer. Biomedicines.

[134] Song F., Chen F.Y., Wu S.Y., Hu B., Liang X.L., Yang H.Q., Cheng J.W., Wang P.X., Guo W., Zhou J. (2021). Mucin 1 Promotes Tumor Progression through Activating WNT/β-Catenin Signaling Pathway in Intrahepatic Cholangiocarcinoma. J. Cancer.

[135] Bengoa-Vergniory N., Kypta R.M. (2015). Canonical and Noncanonical Wnt Signaling in Neural Stem/Progenitor Cells. Cell. Mol. Life Sci..

[136] Srichai M.B., Zent R. (2010). Integrin Structure and Function. Cell-Extracellular Matrix Interactions in Cancer.

[137] Wozniak M.A., Modzelewska K., Kwong L., Keely P.J. (2004). Focal Adhesion Regulation of Cell Behavior. Biochim. Biophys. Acta—Mol. Cell Res..

[138] Ginestier C., Liu S., Diebel M.E., Korkaya H., Luo M., Brown M., Wicinski J., Cabaud O., Charafe-Jauffret E., Birnbaum D. (2010). CXCR1 Blockade Selectively Targets Human Breast Cancer Stem Cells in Vitro and in Xenografts. J. Clin. Investig..

[139] Zhou Q., Bauden M., Andersson R., Hu D., Marko-Varga G., Xu J., Sasor A., Dai H., Pawłowski K., Said Hilmersson K. (2020). YAP1 Is an Independent Prognostic Marker in Pancreatic Cancer and Associated with Extracellular Matrix Remodeling. J. Transl. Med..

[140] Wei C., Wang Y., Li X. (2018). The Role of Hippo Signal Pathway in Breast Cancer Metastasis. OncoTargets Ther..

[141] Gaspar P., Tapon N. (2014). Sensing the Local Environment: Actin Architecture and Hippo Signalling. Curr. Opin. Cell Biol..

[142] Lavado A., Park J.Y., Paré J., Finkelstein D., Pan H., Xu B., Fan Y., Kumar R.P., Neale G., Kwak Y.D. (2018). The Hippo Pathway Prevents YAP/TAZ-Driven Hypertranscription and Controls Neural Progenitor Number. Dev. Cell.

[143] Edwards A., Brennan K. (2021). Notch Signalling in Breast Development and Cancer. Front. Cell Dev. Biol..

[144] Wang X., Bo J., Bridges T., Dugan K.D., Pan T.C., Chodosh L.A., Montell D.J. (2006). Analysis of Cell Migration Using Whole-Genome Expression Profiling of Migratory Cells in the Drosophila Ovary. Dev. Cell.

[145] Hofseth L.J., Hussain S.P., Harris C.C. (2004). P53: 25 Years after Its Discovery. Trends Pharmacol. Sci..

[146] Mellman I., Yarden Y. (2013). Endocytosis and Cancer. Cold Spring Harb. Perspect. Biol..

[147] Hoque M., Elmaghrabi Y.A., Köse M., Beevi S.S., Jose J., Meneses-Salas E., Blanco-Muñoz P., Conway J.R.W., Swarbrick A., Timpson P. (2020). Annexin A6 Improves Anti-Migratory and Anti-Invasive Properties of Tyrosine Kinase Inhibitors in EGFR Overexpressing Human Squamous Epithelial Cells. FEBS J..

[148] Wang C.Y., Lin C.F. (2014). Annexin A2: Its Molecular Regulation and Cellular Expression in Cancer Development. Dis. Markers.

[149] Lee K.M., Nam K., Oh S., Lim J., Kim R.K., Shim D., Choi J.H., Lee S.J., Yu J.H., Lee J.W. (2015). ECM1 Regulates Tumor Metastasis and CSC-like Property through Stabilization of β-Catenin. Oncogene.

[150] Yu H., Königshoff M., Jayachandran A., Handley D., Seeger W., Kaminski N., Eickelberg O. (2008). Transgelin Is a Direct Target of TGF-β/Smad3-dependent Epithelial Cell Migration in Lung Fibrosis. FASEB J..

[151] Lin Y., Buckhaults P.J., Lee J.R., Xiong H., Farrell C., Podolsky R.H., Schade R.R., Dynan W.S. (2009). Association of the Actin-Binding Protein Transgelin with Lymph Node Metastasis in Human Colorectal Cancer. Neoplasia.

[152] Gimona M., Kaverina I., Resch G.P., Vignal E., Burgstaller G. (2003). Calponin Repeats Regulate Actin Filament Stability and Formation of Podosomes in Smooth Muscle Cells. Mol. Biol. Cell.

[153] Lee K.C., Kuo H.C., Shen C.H., Lu C.C., Huang W.S., Hsieh M.C., Huang C.Y., Kuo Y.H., Hsieh Y.Y., Teng C.C. (2017). A Proteomics Approach to Identifying Novel Protein Targets Involved in Erinacine A–Mediated Inhibition of Colorectal Cancer Cells’ Aggressiveness. J. Cell. Mol. Med..

[154] Wang Y., Liu S., Zhang Y., Yang J. (2019). Myosin Heavy Chain 9: Oncogene or Tumor Suppressor Gene?. Med. Sci. Monit..

[155] Mousavi S., Safaralizadeh R., Hosseinpour-Feizi M., Azimzadeh-Isfanjani A., Hashemzadeh S. (2018). Study of Cofilin 1 Gene Expression in Colorectal Cancer. J. Gastrointest. Oncol..

[156] Song Y., Ma X., Zhang M., Wang M., Wang G., Ye Y., Xia W. (2020). Ezrin Mediates Invasion and Metastasis in Tumorigenesis: A Review. Front. Cell Dev. Biol..

[157] Tentler D., Lomert E., Novitskaya K., Barlev N.A. (2019). Role of ACTN4 in Tumorigenesis, Metastasis, and EMT. Cells.

[158] Zhang Y., Luo X., Lin J., Fu S., Feng P., Su H., He X., Liang X., Liu K., Deng W. (2020). Gelsolin Promotes Cancer Progression by Regulating Epithelial-Mesenchymal Transition in Hepatocellular Carcinoma and Correlates with a Poor Prognosis. J. Oncol..

[159] Barnawi R., Al-Khaldi S., Bakheet T., Fallatah M., Alaiya A., Ghebeh H., Al-Alwan M. (2020). Fascin Activates β-Catenin Signaling and Promotes Breast Cancer Stem Cell Function Mainly Through Focal Adhesion Kinase (FAK): Relation With Disease Progression. Front. Oncol..

[160] Beghein E., Devriese D., Van Hoey E., Gettemans J. (2018). Cortactin and Fascin-1 Regulate Extracellular Vesicle Release by Controlling Endosomal Trafficking or Invadopodia Formation and Function. Sci. Rep..

[161] Liu Z., Ning G., Xu R., Cao Y., Meng A., Wang Q. (2016). Fscn1 Is Required for the Trafficking of TGF-β Family Type i Receptors during Endoderm Formation. Nat. Commun..

[162] Liu H., Zhang Y., Li L., Cao J., Guo Y., Wu Y., Gao W. (2021). Fascin Actin-Bundling Protein 1 in Human Cancer: Promising Biomarker or Therapeutic Target?. Mol. Ther.—Oncolytics.

[163] Frame M.C., Patel H., Serrels B., Lietha D., Eck M.J. (2010). The FERM Domain: Organizing the Structure and Function of FAK. Nat. Rev. Mol. Cell Biol..

[164] Wu J.C., Chen Y.C., Kuo C.T., Yu H.W., Chen Y.Q., Chiou A., Kuo J.C. (2015). Focal Adhesion Kinase-Dependent Focal Adhesion Recruitment of SH2 Domains Directs SRC into Focal Adhesions to Regulate Cell Adhesion and Migration. Sci. Rep..

[165] Schlaepfer D.D., Hanks S.K., Hunter T., Geer P. (1994). Van Der Integrin-Mediated Signal Transduction Linked to Ras Pathway by GRB2 Binding to Focal Adhesion Kinase. Nature.

[166] Lim S.T.S. (2013). Nuclear FAK: A New Mode of Gene Regulation from Cellular Adhesions. Mol. Cells.

[167] Mizuno K. (2013). Signaling Mechanisms and Functional Roles of Cofilin Phosphorylation and Dephosphorylation. Cell. Signal..

[168] Garrido-Casado M., Asensio-Juárez G., Vicente-Manzanares M. (2021). Nonmuscle Myosin II Regulation Directs Its Multiple Roles in Cell Migration and Division. Annu. Rev. Cell Dev. Biol..

[169] Schaller M.D., Hildebrand J.D., Shannon J.D., Fox J.W., Vines R.R., Parsons J.T. (1994). Autophosphorylation of the Focal Adhesion Kinase, Pp125FAK, Directs SH2-Dependent Binding of Pp60src. Mol. Cell. Biol..

[170] Polte T.R., Hanks S.K. (1995). Interaction between Focal Adhesion Kinase and Crk-Associated Tyrosine Kinase Substrate P130Cas. Proc. Natl. Acad. Sci. USA.

[171] Chen H.C., Appeddu P.A., Isoda H., Guan J.L. (1996). Phosphorylation of Tyrosine 397 in Focal Adhesion Kinase Is Required for Binding Phosphatidylinositol 3-Kinase. J. Biol. Chem..

[172] Calalb M.B., Polte T.R., Hanks S.K. (1995). Tyrosine Phosphorylation of Focal Adhesion Kinase at Sites in the Catalytic Domain Regulates Kinase Activity: A Role for Src Family Kinases. Mol. Cell. Biol..

[173] Calalb M.B., Zhang X., Polte T.R., Hanks S.K. (1996). Focal Ahdhesion Kinase Tyrosine-861 Is a Major Site of Phosphorylation by Src. Biochem. Biophys. Res. Commun..

[174] Thomas J.W., Ellis B., Boerner R.J., Knight W.B., White G.C., Schaller M.D. (1998). SH2- and SH3-Mediated Interactions between Focal Adhesion Kinase and Src. J. Biol. Chem..

[175] Moriyama K., Iida K., Yahara I. (1996). Phosphorylation of Ser-3 of Cofilin Regulates Its Essential Function on Actin. Genes Cells.

[176] Bamburg J.R. (1999). Proteins of the ADF/Cofilin Family: Essential Regulators of Actin Dynamics. Annu. Rev. Cell Dev. Biol..

[177] Wendt T., Taylor D., Trybus K.M., Taylor K. (2001). Three-Dimensional Image Reconstruction of Dephosphorylated Smooth Muscle Heavy Meromyosin Reveals Asymmetry in the Interaction between Myosin Heads and Placement of Subfragment 2. Proc. Natl. Acad. Sci. USA.

[178] Dulyaninova N.G., House R.P., Betapudi V., Bresnick A.R. (2007). Myosin-IIA Heavy-Chain Phosphorylation Regulates the Motility of MDA-MB-231 Carcinoma Cells. Mol. Biol. Cell.

[179] Beausoleil S.A., Villén J., Gerber S.A., Rush J., Gygi S.P. (2006). A Probability-Based Approach for High-Throughput Protein Phosphorylation Analysis and Site Localization. Nat. Biotechnol..

